# Moving Mendelian Randomization From Traditional Risk Factors to Molecular Targets for Drug Development and Clinical Trials in Nephrology

**DOI:** 10.1016/j.ekir.2026.106350

**Published:** 2026-02-12

**Authors:** Abigail J. Berube, Eryn Yu, Pukhraj S. Gaheer, Matthew B. Lanktree

**Affiliations:** 1 Department of Medicine, McMaster University, Hamilton, Ontario, Canada; 2 Division of Nephrology, St. Joseph’s Healthcare Hamilton, Hamilton, Ontario, Canada; 3 Department of Health Research Methods, Evidence, and Impact, McMaster University, Hamilton, Ontario, Canada; 4 Population Health Research Institute, Hamilton, Ontario, Canada

**Keywords:** drug targets, genomics, Mendelian randomization, multiomics, nephrology, therapeutic discovery

## Abstract

Mendelian randomization leverages the random assortment of alleles at conception to investigate how genetically mediated changes in an exposure affect an outcome while minimizing concerns related to reverse causation and unmeasured confounding. Initially applied to assess the causal impact of modifiable traditional risk factors as mediators of disease risk, Mendelian randomization studies now incorporate large-scale multiomic datasets providing valuable insights for drug target discovery. By analyzing *cis* genetic changes that affect gene activity or protein levels—using advancing techniques like single-cell sequencing and proteomics—Mendelian randomization can identify new therapeutic targets, predict drug target efficacy and effect size before trial development, anticipate adverse effects, reduce late-stage trial failures, and identify opportunities for drug repurposing. This review explains the basic principles, broad applications, and inherent limitations of Mendelian randomization in drug-target identification, validation, and repurposing within the context of kidney disease. Many retrospective examples of concordant conclusions from clinical trials and Mendelian randomization studies have been reported including statins, allopurinol, sodium-glucose cotransporter-2 (SGLT2) inhibitors, and glucagon-like peptide-1 (GLP-1) receptor agonists. Genetic evidence should now be prospectively evaluated for all drugs attempting to traverse the “translational valley of death” in drug development. We summarize current examples, spotlight emerging analytic methodologies such as phenome-wide Mendelian randomization and integrated multiomics, and discuss future directions to accelerate drug development in nephrology.

Kidney disease ranks among the leading causes of death in the 21st century, accounting for 1.2 million deaths globally in 2017.^1^ An estimated 753 million individuals live with chronic kidney disease (CKD) worldwide.^2^ With the aging global population and the rising prevalence of diabetes mellitus and heart disease, CKD is projected to become the most common chronic illness by 2040.^3^ Despite the high mortality associated with CKD from both kidney failure and cardiovascular disease, development of effective treatments has been challenging. Yet, there have been recent successes leading to 4 pillars of treatment: renin-angiotensin-aldosterone system (RAAS) inhibition, sodium-glucose cotransporter-2 (SGLT2) inhibitors, non-steroidal mineralocorticoid receptor antagonists, and glucagon-like peptide-1 (GLP-1) receptor agonists.^4^ Notably, none of these therapies are targeted specifically to CKD pathophysiology, nor were developed directly for treating CKD.

Drug development is an expensive and complex process requiring knowledge of disease pathogenesis and modifiers, insights from cellular and animal models, and data from genetics and biomarkers in humans, all before clinical trials in humans are considered. Many putative therapies are lost in the “translational valley of death” in drug development, which is the gap between basic scientific findings and its application to human disease.^5^ Amongst the drug candidates that do traverse the “translational valley of death,” there is significant attrition because of failures in the clinical trials. Success rates across clinical trial phases vary from 31% to 68%, with phase II trials demonstrating the lowest success rate.^6^ The inability to bring new drugs to market is particularly profound in the field of nephrology, which accounted for less than 1% of novel drug approvals between 2016 and 2021 across therapeutic areas.^7^

Rather than simply repositioning or repurposing therapies, new therapeutic strategies can be discovered with translational research techniques using high-throughput data sources, including DNA sequencing, gene and protein expression, biomarker measurement, and multiomic strategies incorporating data from multiple levels of the “central dogma of molecular biology” (i.e., DNA → RNA → protein). These multiomic techniques represent a generational development in generating evidence to support new drug targets. The addition of Mendelian randomization to the drug discovery workflow can provide a foundational assessment of the therapeutic potential and safety profile of drug targets at early stages of research and development process prioritizing candidates for animal or cellular model investigations, as well as prioritizing targets for shifting into human trials.^8^

### Overview of Mendelian Randomization

Mendelian randomization seeks to determine if a genetically induced change in exposure is associated with an outcome (Figure 1). A glossary of terms used in Mendelian randomization is provided in Table 1. Mendelian randomization utilizes Mendel’s “law of independent assortment” which states that alleles for different traits assort independently of each other at gamete formation. Simply put, a pea plant having a wrinkled or round seed is independent from whether it has a purple or white flower. Akin to “nature’s randomized controlled trial,” whether an individual has brown or blue eyes is independent of whether they have relatively higher or lower uromodulin in their blood, and also independent of their average daily calorie intake. The genotype one receives is free from impact of subsequent disease and is constant throughout life removing reverse causation and allowing for directional causal inferences.^9^ This is in contrast to observational association studies where the direction of effect cannot be determined (i.e., does hypertension cause CKD or does CKD cause hypertension?) Thus, if a genetic variant or set of variants influences an exposure, such as a trait, risk factor, or biomarker, we can estimate how genetically higher or lower levels of that exposure relate to subsequent disease risk.

**Figure 1:**
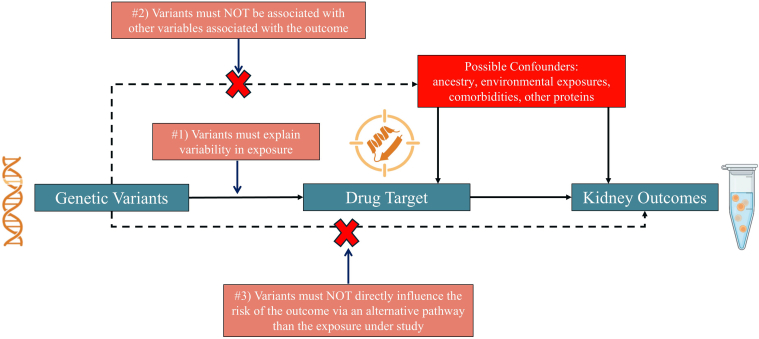
Conceptual framework for drug-target Mendelian Randomization, to evaluate potential causal effects of genetically altered drug target action on chronic kidney disease. Naturally occurring genetic variation serves as a source of quasi-randomized perturbation of drug targets, analogous to treatment allocation in a randomized controlled trial. Solid lines indicate causal pathways that are hypothesized to exist, whereas the dashed lines indicate pathways that are hypothesized not to exist forming the following 3 core Mendelian randomization assumptions: (i) the relevance assumption;(ii) the independence assumption; and (iii) the exclusion restriction assumption.

**Table 1:** Glossary of terms used in Mendelian randomization

Term	Definition
Allele	Alternate version of a genetic sequence at a specific genomic location or genetic variant. Individuals inherit 1 allele from each parent.
Central dogma of molecular biology	The flow of genetic information where DNA is transcribed into RNA and translated into protein. This framework explains how genetic variants influence all molecular traits.
Colocalization	A statistical method used to evaluate whether two traits share a causal variant within a genomic region, rather than being driven by distinct but correlated variants. Traits pairs can be physical, like height and weight, or molecular, like eQTL and eGFR.
eQTL	Genetic variant associated with differences in quantity of gene expression (mRNA).
Gene	A segment of DNA that, through a distinct sequence of nucleotides, encodes a functional product, such as a protein or regulatory RNAs.
GWAS	A study design that uses large populations to assess the association of common genetic variants across the genome with a trait. Traits can be physical – like height or blood pressure – or molecular like a protein concentration.
Instrumental variable analysis	A variable used to infer the causal effect of an exposure on an outcome by serving as a proxy for the exposure. An instrumental variable must influence the outcome only through its effect on the exposure and not through alternative pathways. In Mendelian randomization, genetic variants are used as the instrumental variables.
LD	The non-random correlation between alleles because of shared inheritance driven by physical proximity on the chromosome. LD complicates genetic analyses because multiple variants can appear associated with a trait even when only 1 is causal.
Mendelian randomization	An analytical approach that uses genetic variants as instrumental variables to estimate the causal effect of an exposure on an outcome, leveraging natural randomization of alleles at fertilization to reduce confounding and reverse causation.
Mendelian randomization sensitivity analyses	Analytical methods used to assess the robustness of Mendelian randomization findings and detect and adjust for violations of assumptions including horizontal pleiotropy.
PheWAS	A study design that tests the association between a single genetic variant (or polygenic score) and a broad set of phenotypes (the “phenome”), useful for detecting pleiotropy and identifying on-target adverse effects.
Pleiotropy	A phenomenon in which a single genetic variant influences two or more unrelated traits. Pleiotropy can be classified as vertical (whereby the effects occur through the same biological pathway) or horizontal (the effects occur via unrelated pathways).
pQTL	Genetic variants associated with differences in protein levels. pQTL are frequently used for Mendelian randomization studies that model proteins as causal mediators or drug targets.
PWAS	Also known as Proteome-wide Mendelian randomization study or proteogenomic study, a study design that uses large populations to assess the impact of genetically predicted changes in each protein’s quantity on a given trait. Tests for a correlation between the effect of each genetic variant on protein concentration and its impact on a trait of interest.
Reverse causation	A form of bias that occurs when the outcome influences the exposure, rather than the exposure influencing the outcome.
TWAS	A study design that uses large populations to assess the impact of genetically predicted changes in each gene’s expression on a given trait. Effectively tests for a correlation between the effect size of each genetic variant on the quantity of a gene’s expression and its impact on the trait of interest.
Weak instrument	A genetic instrument that explains only a small proportion of the variance in the exposure. Weak instruments can bias Mendelian randomization estimates, particularly in 1-sample Mendelian randomization, and reduce statistical power.

eGFR, estimated glomerular filtration rate; eQTL, expression quantitative trait loci; GWAS, genome-wide association study; LD, linkage disequilibrium; PheWAS, phenome-wide association study; pQTL, protein quantitative trait loci; PWAS, proteome-wide association study; TWAS, transcriptome- wide association study.

For Mendelian randomization to work there needs to be an adequate proportion of variability in the exposure explained by known genetic variants (i.e., the exposure must be heritable and we know the variants contributing to that heritability). Certainly Mendelian randomization is not a panacea, and has well described and studied assumptions including the following: (i) absence of pleiotropy (i.e., the genetic variant cannot impact the likelihood of the outcome via a separate pathway than the intermediate biomarker); (ii) absence of linkage disequilibrium (variants physically close to each other on the chromosome are inherited together more often and can lead to double counting of effects); (iii) minimal measurement error in exposure or outcome; and (iv) lack of variability in the genetic effect on the exposure based on time of the exposure and outcome measurement.

Using 2 different study samples, 1 to identify and evaluate the effect size of the genetic variants on the exposure, and a second sample to evaluate the effect of the same variants on the outcome—known as “2 sample” Mendelian randomization—is now standard practice and reduces the possibility of bias compared to one sample designs. Using 2 sample Mendelian randomization, the expectation is that a weak instrument (i.e., when genetic variants explain a very small proportion of the heritability of the exposure leading to low power) will bias results towards the null reducing false positives.

Mendelian randomization plots visually display the association of each genetic variant with the exposure and the outcome (Figure 2). In these plots, each point represents a single genetic variant, with its position on the x-axis showing the effect on the exposure and its position on the y-axis showing the effect on the outcome. A regression line is drawn, minimizing the distance between the points and the line. The regression line is anchored through the origin, as a variant with no effect on the exposure should have no effect on the outcome. The slope of this regression line gives the causal effect on the outcome for a given change in exposure. The statistical significance (*P*-value) of the Mendelian randomization analysis indicates how strongly the genetically altered exposure is associated with the outcome, a combination of the effect size of the genetically altered exposure on the outcome and how close the points are to the regression line. Thus, a Mendelian randomization *P*-value of 0.05 indicates a 5% chance of seeing an effect at least as large as the one observed if the “true” causal effect was actually zero.

**Figure 2:**
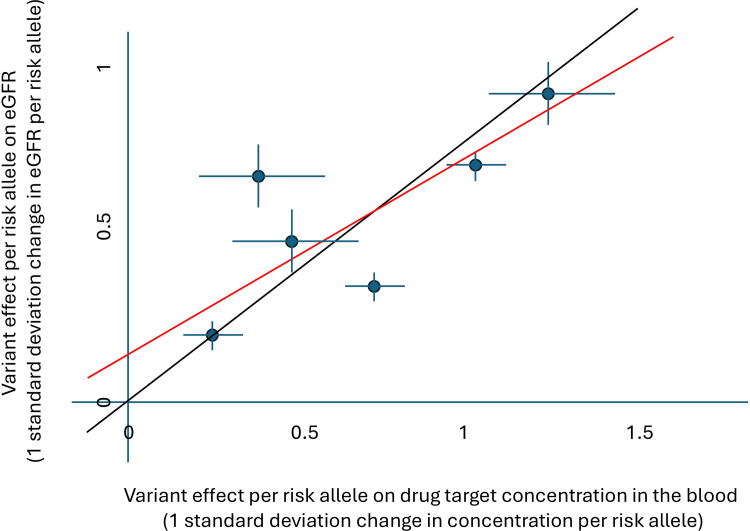
Mendelian randomization plot. Each point reflects a single genetic variant. The position of the point on the x-axis represents the effect of the variant on the exposure (in this case drug target concentration in the blood) with confidence intervals drawn, and the position of the point on the y-axis represents the effect of the variant on the outcome (in this case eGFR) with confidence intervals drawn. The Mendelian randomization causal estimate is represented by the slope of the black regression line. In MR-Egger, the regression line is not constrained to go through the origin, and is drawn in red. eGFR, estimated glomerular filtration rate.

Statistical techniques and sensitivity analyses to detect and attempt to adjust or correct for pleiotropy have been developed including inverse variance weighting, weighted median estimator, “MR-Egger” regression, “Mendelian Randomization Pleiotropy RESidual Sum and Outlier (MR-PRESSO)”, and multivariable Mendelian randomization among others. Inverse variance weighting gives greater weight to variants with greater precision in the estimate of the effect on the exposure. The weighted median estimator reports the median causal effect estimate, effectively excluding outlier causal effect estimates from outlier variants yielding a true estimate even in the presence of many biased variants.^10^^,^^11^ In “MR-Egger” the Mendelian randomization regression line isn’t forced to go through the origin, allowing for detection of an upward or downward tendency in the genetic effect on the outcome even as genetic effects on the exposure approach zero, but results in a substantial loss of power. The “MR-PRESSO” method detects and removes outlier variants that are associated with other traits that may represent an alternative way for the variant to impact the outcome.^12^ Multivariable Mendelian randomization attempts to adjust for the effect of the variants on multiple exposures.^13^ For example, when evaluating the effect of a variant on CKD, in multivariable Mendelian randomization one could attempt to adjust for a known effect of a variant on diabetes risk. Further details of these methods and their limitations are beyond the scope of this review but an extensive and growing literature exists.

Initially used to evaluate traditional risk factors such as lipid levels,^14^ body mass index,^15^ or hypertension,^16^ Mendelian randomization is an even more effective methodology for assessing causal relationships between biomarkers or molecular traits in multiomic networks for the following reasons: (i) a larger proportion of variability in gene expression or biomarker concentration is explained by genetic variants near the gene coding for the protein compared to complex heterogeneous quantitative traits, (ii) genetic variants near a gene directly impacting gene expression are less likely to impact numerous biological pathways than selecting variants throughout the genome impacting a complex trait like hypertension, and (iii) the ability of therapies such as RNA interference or monoclonal antibodies to alter gene expression or protein quantity or activity in a targeted fashion more closely parallels the Mendelian randomization analysis.

Drug target Mendelian randomization uses genetic variants in close proximity to genes encoding the target called *cis* genetic variants typically within 50–500 kb of the gene. Drug target Mendelian randomization studies typically employ the following 4 strategies: (i) identify new drug targets; (ii) evaluate the potential efficacy of an already identified drug target on multiple exposures; (iii) screen a drug target for adverse effects; or (iv) identify unrecognized benefits that may be a new indication for repurposing (Figure 3).^17^ Guidelines for performing and interpreting Mendelian randomization studies have previously been published,^18^ as have sample papers for drug target, proteogenomic, and proteome-wide Mendelian randomization.^19^, ^20^, ^21^

**Figure 3:**
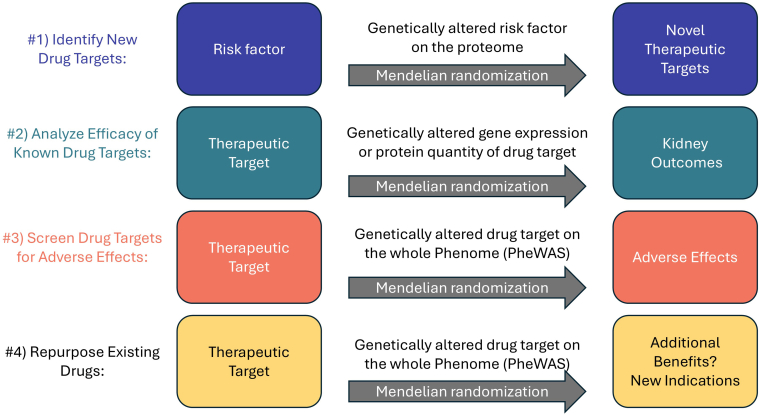
Four applications of Mendelian randomization to drug target development. PheWAS, phenome-wide association study

### Nephrology Publications Using Mendelian Randomization

We reviewed all nephrology Mendelian randomization articles between 2014 to 2023 (Supplementary Methods and Figure S1). After screening 408 articles, 246 studies were included in our review. 214 (87%) of the included nephrology Mendelian randomization studies investigated a kidney outcome, and 57 (23%) examined kidney disease as an exposure. The number of nephrology-related Mendelian randomization studies increased from 3 in 2014 to 91 in 2023 (Figure 4). The first Mendelian randomization study evaluating a drug target was reported in 2016, and only 25 drug target nephrology Mendelian randomization studies have been published up to 2023. Only 4 nephrology drug target Mendelian randomization studies performed druggability assessments. Studies including multiomic approaches grew from 0 in 2014–2017 to 15 in 2023, a number that is sure to continue to rise.

**Figure 4:**
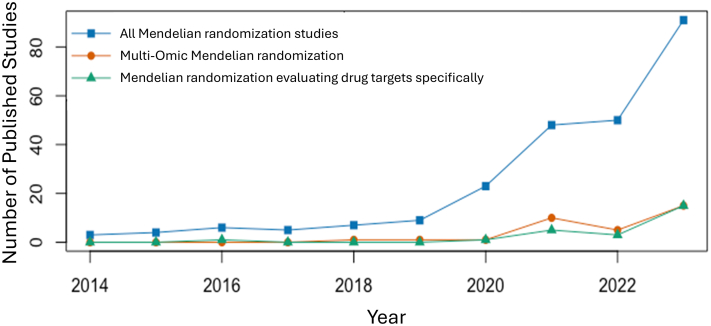
Growth of Mendelian randomization in nephrology from 2014 to 2023.

### Searching for Novel Therapeutic Targets

Proteogenomic or proteome-wide Mendelian randomization studies evaluate the impact of genetically mediated changes to all measurable biomarkers in the genome. Using multiancestry proteome-wide Mendelian randomization, we evaluated the impact of genetically predicted alterations of 1161 biomarkers in 12,066 participants from the multiethnic Prospective Urban and Rural Epidemiological (PURE) study.^21^ We then examined the correlation between genetic effects on protein concentration and genetic effects on eGFR among UK biobank and CKD genetics consortium (CKDGen) participants. Uromodulin (*UMOD*) arose as a positive control for the approach, but 22 biomarkers were identified as causal mediators of eGFR regulation including biologically relevant genes identified in genome-wide associations studies (i.e., inhibin beta C [*INHBC*] and collagen type 18 alpha 1 [*COL18A1*]), but also new genes without prior support from genome-wide genetic studies (i.e., renal dipeptidase 1 [*DPEP1*]).

Multiomic Mendelian randomization provides a systematic network approach for the discovery of drug targets by leveraging genetic associations across multiple molecular layers, such as the transcriptome, proteome, methylome, and metabolome, to identify causal links between these molecular traits and disease outcomes.^22^, ^23^, ^24^, ^25^ Multiomic Mendelian randomization leverages variants associated with gene expression known as expression quantitative trait loci (eQTL), or protein quantity known as protein quantitative trait loci (pQTL). High throughput biomarker measurements can arise from either aptamer-based or antibody-based protein biomarker quantification approaches.^26^ eQTL can arise out of samples from whole blood from sources such as the eQTL genetics consortium (eQTLGen),^27^ but more recently tissue compartment-specific and cell-specific eQTL data is preferred from single cell sequencing.^28^ By using eQTLs or pQTLs as instruments, omic-wide Mendelian randomization tests the causal effect of altered genetically predicted gene expression or protein abundance of thousands of targets on disease outcomes in a hypothesis-free manner, allowing for the simultaneous evaluation of numerous potential targets, enabling the discovery of novel therapeutic avenues.^29^, ^30^, ^31^ Comparison of the results of a single target to the distribution of all targets also facilitates multiple testing correction and reduction in false discovery rates, akin to shifting from candidate gene studies to genome-wide analyses.

These multiomic approaches require large, often population-scale, well-phenotyped datasets, but large genome-wide association study (GWAS) meta-analyses, such as CKDGen, biomarker and expression datasets, such as The Human Protein Atlas, and biobank data, such as the UK biobank, the All of Us biobank, and many more have been developed.^32^ Tissue-specificity in gene expression or protein abundance, where the genetic regulation of gene or protein expression varies across tissues, is unveiling a whole new level for Mendelian randomization analyses.^33^^,^^34^ For example, while it is generally believed a correlation exists (i.e., a variant that increases gene expression compared to wild type in one cell type is unlikely to reduce expression of the same gene in a different cell type), an eQTL identified in whole blood may not reflect changes in gene expression in kidney interstitial cells or podocytes. Thus, while early efforts were largely limited to whole blood with adequate sample sizes, development of high-quality “omic” data for relevant tissues is rapidly progressing. Proteomic data from kidney tissues is relatively scarce (not typically available in many thousands of individuals) because of the challenges associated with performing kidney biopsies.^35^ This lack of kidney-specific proteomic data is especially pronounced in non-European populations.^36^

Another strategy for drug target identification is to evaluate the impact of genetically predicted changes in a risk factor on the whole proteome. For example, diabetes and diabetic nephropathy are major contributors to CKD. The impact of genetically predicted changes in glucose on the concentration of all measurable proteins in the proteome could identify causal mediators of glucose induced kidney injury.

### Assessing the Efficacy of Therapeutic Targets

Once a therapeutic target is identified, preclinical cell and animal models are employed to further validate targets. The opposite can also occur, where targets identified from knowledge of pathophysiology or models can be evaluated as a candidate in multiomic data. However, after making the leap across the “valley of death” up to 52% of drug candidates fail in phase II or phase III clinical trials because of lack of clinical efficacy,^37^ underscoring the limitations of traditional methods like *in vitro* studies and animal models, which often poorly predict human outcomes, including kidney outcomes.^38^^,^^39^ Drugs with genetic evidence supporting their efficacy may be up to twice as likely to succeed in clinical trials than those without genetic evidence.^40^ Mendelian randomization analyses that retrospectively assess the efficacy of major therapeutic classes approved for delaying eGFR decline and CKD progression generally show strong agreement with evidence from randomized controlled trials (Table 2). This concordance between Mendelian randomization estimates and clinical trial outcomes across established kidney protective therapies supports the growing view that Mendelian randomization can anticipate the effect observed in future clinical studies.

**Table 2:** Comparing results of Mendelian randomization analysis to results of randomized controlled trials in nephrology

Drug Class	Mendelian randomization	Trial results	Concordance
SGLT2 inhibitors	*SLC5A2* variants as proxy for *SGLT2* inhibition demonstrates protective effects on eGFR and reduced albuminuria.^41^, ^42^, ^43^	SGLT2 inhibitors delay eGFR loss and CKD progression.^44^, ^45^, ^46^, ^47^, ^48^, ^49^, ^50^	Yes
ACE inhibitors	ACE variants are associated with improved eGFR.^51^	ACE inhibitors slow eGFR decline and delays progression to kidney failure.^52^, ^53^, ^54^	Yes
GLP-1 receptor agonists	Higher *GLP1R* expression is associated with lower hemoglobin A_1_C, lower body weight, higher eGFR, and slower CKD progression.^43^^,^^55^^,^^56^	GLP-1 receptor agonists reduce kidney outcomes and attenuate eGFR decline.^57^, ^58^, ^59^, ^60^	Yes
Statins	*HMGCR* variants are not associated with attenuated eGFR decline.^61^, ^62^, ^63^, ^64^	Meta-analyses show no protective effect of statin use on eGFR decline.^65^, ^66^, ^67^, ^68^	Yes
Urate lowering therapy	No causal effects of variants impacting serum urate on eGFR, albuminuria, nor risk of CKD.^69^	Allopurinol and febuxostat reduced serum urate, but do not slow eGFR decline.^70^^,^^71^	Yes
Endothelin inhibition	Genetically altered endothelial nitric oxide synthase pathway activation correlates with higher eGFR and lower risk of CKD.^72^	Selective endothelin receptor antagonists protect against kidney outcome but limited by adverse events.^73^	Yes
Nonsteroidal mineralocorticoid receptor antagonist	Genetically predicted renin-independent hyperaldosteronism is causally associated with eGFR and a higher risk of CKD.^74^	In patients with CKD and type 2 diabetes mellitus, finerenone reduced the risk of CKD progression and cardiovascular events.^75^	Yes
Thiazide diuretics	Thiazide-sensitive sodium chloride cotransporter (*SLC12A3*) variants had consistent effects on systolic blood pressure and kidney stone risk.^76^	Among patients with recurrent kidney stones, recurrence did not differ with thiazide diuretics.^77^	No

ACE, angiotensin-converting enzyme; CKD, chronic kidney disease; eGFR, estimated glomerular filtration rate; GLP-1, glucagon-like peptide-1; SGLT2, sodium-glucose cotransporter-2.

### Screening for Adverse Effects Using Phenome-Wide Mendelian Randomization

A comprehensive understanding of a drug target’s safety profile is crucial in drug development. The initial “first-in-human” phase I studies examine treatment tolerability, identify safe dose ranges, evaluate pharmacokinetics, and look for common side effects. Even the largest phase III studies fail to identify adverse effects that are rare or arise from long-term use.^78^ As many as 24% of phase II or III clinical trial failures are attributed to unanticipated toxic effects in humans, and many adverse effects arise even after trials are done.^37^ Animal models, the standard approach for preliminary toxicity screening in preclinical trials, often yield results with a low negative predictive value for human toxicity (i.e., the absence of toxic effects in animal models does not provide strong evidence that the drug will not cause harmful side effects in humans),^79^ and may predict as low as 19% of postmarket serious adverse reactions.^80^ Moreover, animal models lack common comorbidities limiting their relevance. Mendelian randomization can play a critical role when screening for adverse effects of a treatment.

A phenome-wide association study (PheWAS), pronounced “FEE-wahss”, is a study design that tests the association of a set of variants with a wide expanse of phenotypes – the “phenome.” When used in combination with Mendelian randomization, we can test the correlation of the effect of genetic variants on a therapeutic target with the effect of the same genetic variants on a wide range of phenotypes, allowing researchers to identify potential adverse effects.^81^^,^^82^ Phenome-wide Mendelian randomization tests the causal effect of the genetic proxies of the novel drug on risk of developing hundreds (or more) adverse events.^83^

However, using Mendelian randomization to screen therapeutic targets for adverse effects shares the same limitations as assessing the efficacy of therapeutic targets. There need to be genetic variants associated with risk of the adverse event. There are no genetic instruments for risk of allergic events, anaphylaxis, or Stevens-Johnson syndrome, thus Mendelian randomization cannot be used to evaluate the risk of these reactions. On-target adverse events are those mediated directly by the exposure evaluated in the Mendelian randomization analysis. For example, reducing *UMOD* expression may reduce CKD progression in those with uromodulin mediated autosomal dominant tubulointerstitial kidney disease (ADTKD-UMOD), but the reduction in uromodulin may also increase risk of urinary infections or kidney stones. Off-target effects are the result of horizontal pleiotropy, where the adverse effects arise from modulation of other biological targets unrelated to the primary exposure of interest.^84^ In this example, a variant impacting *UMOD* expression may also alter expression of an adjacent gene impacting a different phenotype.

Early detection of potential safety concerns could allow for curating data collection in clinical trials, reduce the likelihood of late-stage clinical trial failures or postmarketing withdrawals, as well as minimizing patient exposure to harmful drug side effects in clinical trials.^85^

### Repurposing Existing Drugs for Novel Indications

Repurposing drugs circumvents major challenges in drug discovery as safety profiles and optimal dosing and formulation are already established. Repurposing shortens development timelines and can save billions of dollars compared to the costs associated with developing an entirely new drug. Repurposing has proven to be an effective strategy for developing new treatments for CKD including SGLT2 inhibitors and GLP1-receptor agonists.^86^

Mendelian randomization can facilitate the discovery of novel repurposing opportunities. Through examining the effects of genetic proxies for drug targets, Mendelian randomization can be used to test the causal effect of these variants on a wide range of phenotypes outside of the drug’s original indication.^87^ In the same manner phenome-wide Mendelian randomization can identify possible adverse events, it can also identify on target beneficial effects that could be used as an indication for the drug.^88^

For example, thiazide diuretics are primarily used as a first line treatment for blood pressure control, but they have also been used to reduce urinary calcium and kidney stone risk. A high impact clinical trial called the practice into question.^77^ Mendelian randomization using genetic variants near the thiazide-sensitive sodium chloride cotransporter gene (*SLC12A3)* showed a consistent effects of the *SLC12A3* variants on systolic blood pressure and kidney stone risk.^76^ Further clinical studies will be needed to resolve this discordance; to begin with perhaps thiazide diuretics would only be successful in reducing stone risk in individuals with high urinary calcium.

By leveraging existing genetic and clinical data, drug repurposing Mendelian randomization can inform the optimization of the initial phases of drug repositioning clinical trials, increasing the likelihood of successful repurposing efforts, reducing the time and costs associated with such endeavours.^89^ Nonetheless, although Mendelian randomization can provide a rationale for drug repurposing, clinical trials are of course ultimately required to confirm the therapeutic potential in new indications.

### Challenges of Using Mendelian Randomization in Drug Development

With the rise of automated analytic tools and public access to summary-level genome-wide association study data, poor quality Mendelian randomization studies could be performed by pumping a large number of potential risk factors on any outcome of interest resulting in false-positive results and low-quality publications. Certainly, many journals have been inundated with such Mendelian randomization analyses. To improve the reliability of Mendelian randomization, external replication of results in multiple datasets, using many sensitivity analysis techniques, with rational biological hypotheses, triangulation of evidence from multiple phenotypes and experimental designs, and use of both positive and negative controls is essential.

For example, when evaluating adenosine triphosphate citrate lyase on CKD risk, the same genetic instrument should have consistent beneficial effects on low density lipoprotein cholesterol and cardiovascular risk, effectively serving as positive controls for use of the genetic instrument to assess adenosine triphosphate citrate lyase inhibition on kidney outcomes.^90^ Evaluating the association of the genetic instrument on other phenotypes, such as schizophrenia or height, can serve as negative controls, supporting the absence of overfitting, population stratification, or bias in effect estimates.

Selecting targets specific to the drugs action is essential. For example, variants in the *SLCO1B1* gene are associated with statin intolerance, impacting a patient’s likelihood of adhering to statin therapy,^91^ rather than directly influencing statins target of 3-hydroxy-3-methyl-glutaryl -coenzyme A reductase.^92^ Using *SLCO1B1* variants as proxies in Mendelian randomization studies would lead to incorrect conclusions about statins’ effectiveness in reducing cardiovascular risk, as the variants relate more to drug tolerance and adherence rather than target inhibition.

Multiprotein targets, where the drug target is a protein complex made up of several individual protein subunits, presents another challenge. It is difficult to account for subunit interactions and unequal contributions of protein subunits in determining clinical phenotypes.^93^ This is an important confounder to consider when considering novel drugs targeting kidney pathways as many are reliant on ion channels and transporters formed from multisubunit protein complexes.^94^ Situations where there can be compensation from alternative pathways can nullify signals from Mendelian randomization. Activins and inhibins are composed of many homo- and hetero-dimers and increased expression of 1 activin subtype could impact expression of the other subtypes complicating Mendelian randomization analyses.^95^

After a novel therapeutic target has been identified, a druggability assessment may be conducted to further characterize the pharmaceutical potential of the discovery. In the context of Mendelian randomization studies, a druggability assessment evaluates the potential of a biological target to be modulated by a small molecule drug, focusing on both the target’s characteristics and its role within biological pathways.^96^ This assessment is essential for determining the viability of therapeutic targets identified through ‘omicwide’ approaches. It involves analyzing the structural features of the target, such as its binding site and overall structure, which influences how effectively a drug can interact with it, and comparing these features to targets of existing drugs to search for similarities that may indicate pharmaceutical effectiveness.^97^ By integrating these factors, druggability assessments help prioritize targets for further validation and experimentation, ensuring that those with the highest potential for successful drug development are advanced through the research pipeline.

Polygenic scores, a rapidly growing area of genetic research, are sometimes conflated with Mendelian randomization despite serving completely different purposes. By design, polygenic risk scores aggregate genome-wide variants across numerous biological pathways to quantify genetic risk, and thus inherently include horizontal pleiotropy, violating core Mendelian randomization assumptions. The deliberate selection of genetic variants focusing on a single exposure of interest is the methodological strength that distinguishes Mendelian randomization from polygenic scores and enables causal inference for drug target evaluation.^9^ However, polygenic risk scores could theoretically be used for risk stratification to identify people at higher risk of outcomes, and thus enrich the sample for outcomes and improve the power of a clinical trial. Further, polygenic response scores could identify population subgroups predicted to exhibit varying treatment responses.

Finally, because Mendelian randomization reflects the lifetime impact of genetic variants, it may not accurately capture the effect of short-acting therapies.^93^ For instance, consider insulin and hyperkalemia.^98^ In a Mendelian randomization study examining genetic variants that influence insulin levels, association of genetically altered insulin and hyperkalemia would reflect the long-term effects of diabetes risk on chronic kidney disease which predisposes to hyperkalemia.^99^ In contrast, insulin’s immediate effect is to cause inward shift of potassium into cells and hypokalemia in the acute setting. Recognizing the many assumptions and limitations of Mendelian randomization is essential to interpret its findings appropriately.

## Conclusion

Mendelian randomization offers significant promise for optimizing the CKD drug discovery and assisting therapies to traverse the “translational valley of death.” By enabling relatively low-cost preclinical analyses, Mendelian randomization supports drug development, including exploration of novel therapeutics, efficacy and safety assessments, and repurposing of existing drugs. These insights can guide strategic decisions on which treatments should be advanced towards further development and reduce the likelihood of clinical trial failures. As multiomic datasets expand in sample size and data types, and computational tools evolve, Mendelian randomization’s role in identifying therapeutic targets and predicting clinical outcomes is poised to grow, transforming the future of drug development.

## Disclosure

MBL has received speaker and advisory fees from Otsuka, GlaxoSmithKline, and Hikma. All other authors have no conflicts of interest to disclose.

## Supplementary Material

Supplementary File (PDF) **Supplementary Methods. Figure S1.** Prisma diagram of literature review.

## Footnotes

Supplementary File (PDF)

**Supplementary Methods.**
**Figure S1.** Prisma diagram of literature review.
