# Supplementary material for: Moving Mendelian Randomization From Traditional Risk Factors to Molecular Targets for Drug Development and Clinical Trials in Nephrology
Source: Kidney Int Rep. 2026 Feb 12;11(5):106350. doi: 10.1016/j.ekir.2026.106350 (PMC12997310; doi:10.1016/j.ekir.2026.106350)

Supplementary methods

The Web of Science database was used to search for Mendelian Randomization studies published between January 1st, 2014 to December 31st 2023 in which at least one of the primary exposures or outcomes was related to renal function or renal health. The search string used was “mendelian randomization” AND (nephr* OR kidney OR CKD OR PKD or glumerul*). Following the database search the Covidence platform was used to conduct article screening and data extraction. Studies were included if MR was a primary analysis method and if the MR included either a kidney outcome or kidney exposure. Preprints, editorials, thesis dissertations, meeting proceedings, abstract-only publications, and non-English studies were excluded. Studies that included cancer as a primary outcome or exposure were also excluded given that MR utilizes germ line mutations to form the instrumental variables and analyze outcome associations and cancer arises from somatic mutations. Article screening was performed in two steps: title and abstract screening and full text screening. Only studies which passed title and abstract screening progressed to full text screening. Two investigators independently performed both screening steps. Disagreements were resolved through discussion between both investigators. Only one investigator was required per individual article data extraction. Data extracted included technical details regarding the MR conducted such as analysis types and data sources, as well as broader details regarding the use of MR within drug target studies. No institutional review board approval was required for this review.

Supplemental Figure S1. Prisma diagram of literature review.

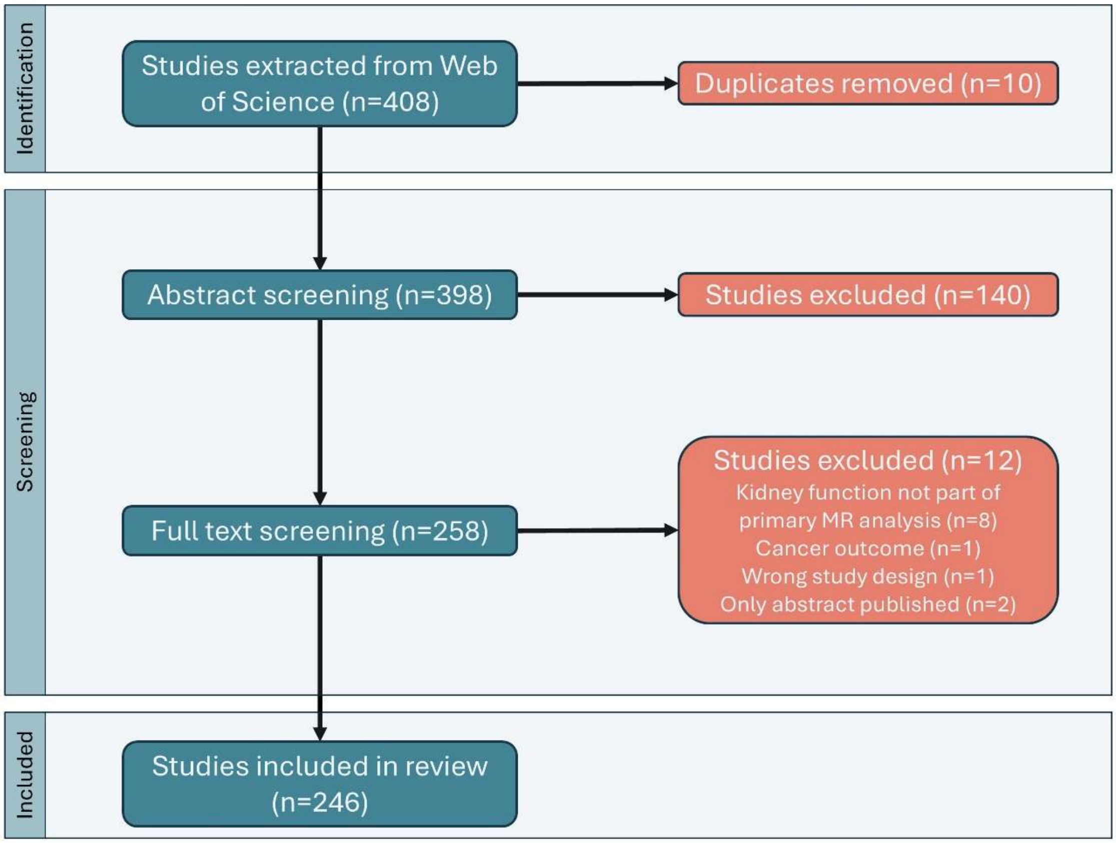

Supplement: Supplementary File (PDF) — Supplementary Methods. Figure S1. Prisma diagram of literature review. [file mmc1.pdf]
